# Scoliosis after cardiac surgery for congenital heart disease: a long-term follow-up study

**DOI:** 10.3389/fcvm.2026.1833617

**Published:** 2026-08-13

**Authors:** Mengqi Zhao, Xuechen Liu, Junxiang Pan, Xinyu Cong, Yongqiang Jin, Lianyi Wang

**Affiliations:** 1 Heart Center, The First Hospital of Tsinghua University, School of Clinical Medicine, Tsinghua Medicine, Tsinghua University, Beijing, China; 2 The First Hospital of Tsinghua University, School of Clinical Medicine, Tsinghua Medicine, Tsinghua University, Beijing, China

**Keywords:** cardiac surgery, congenital heart disease, long-term follow-up, risk factors, scoliosis

## Abstract

**Background:**

As survival among patients with congenital heart disease (CHD) has improved, scoliosis after cardiac surgery has increasingly been recognized. However, the clinical characteristics and risk factors associated with scoliosis severity remain incompletely understood.

**Methods:**

We retrospectively reviewed patients who underwent cardiac surgery for CHD at our center between 2000 and 2022 and conducted long-term follow-up. Patients who developed scoliosis were matched with controls without scoliosis using propensity score matching (PSM). Clinical and radiographic data were analyzed. Logistic regression was used to identify independent risk factors for greater scoliosis severity, and Kaplan–Meier analysis to evaluate cumulative incidence. Associations between cardiac morphology and scoliosis severity were also assessed.

**Results:**

A total of 39 patients who developed scoliosis after CHD surgery and 39 matched patients without scoliosis were included. The median follow-up duration for the overall cohort was 12.75 (10.55–18.15) years. Compared with the non-scoliosis group, patients with scoliosis had a significantly higher cardiothoracic ratio (CTR) [55.48 [51.68–58.81] vs. 52.04 [46.62–55.56], *P* = 0.004]. Among patients with scoliosis, females accounted for 58.97%, and the median age at scoliosis diagnosis was 10.56 (8.54–13.16) years. Single thoracic curves predominated, with the apical vertebra most commonly located at the T8-T9 level. Cardiac surgery before 1 year of age was more common among patients with higher Cobb angles and was associated with earlier scoliosis onset. Patients with higher Cobb angles had a higher CTR than those with lower Cobb angles [58.48 [55.39–61.68] vs. 52.85 [49.25–55.88], *P* = 0.003], and CTR was positively correlated with Cobb angle (r = 0.494, *P* = 0.001). Multivariate logistic regression showed that a higher CTR was independently associated with greater scoliosis severity (OR 4.79, 95% CI 1.01–22.68, *P* = 0.048). Relative leftward extension of the cardiac silhouette was positively correlated with Cobb angle (r = 0.470, *P* = 0.003).

**Conclusions:**

Scoliosis after cardiac surgery for CHD most commonly presented as a right-convex single thoracic curve. A higher CTR was associated with both the presence of scoliosis and greater scoliosis severity. Long-term spinal surveillance should be considered, particularly in high-risk children.

## 1 Introduction

Scoliosis is one of the most common spinal deformities, and its association with congenital heart disease (CHD) has been widely recognized (1). Previous studies have reported that 10%–42% of patients with CHD exhibit varying degrees of scoliosis (2), which is markedly higher than the 2%–3% prevalence in the general adolescent population (3). The mechanisms underlying this increased risk remain unclear and are likely multifactorial. Thoracic surgery has been considered an important contributor to the development of scoliosis. Surgical treatment of CHD may involve thoracotomy, median sternotomy (MS), or a combination of both approaches. Cardiac surgery may disrupt thoracic cage stability, potentially contributing to the development of scoliosis (4). However, the impact of cardiothoracic surgery on the progression and severity of scoliosis in patients with CHD remains uncertain.

Therefore, the present study aimed to systematically analyze the clinical and radiographic characteristics of scoliosis in patients with CHD who underwent cardiac surgery at our institution, and to further explore potential risk factors associated with scoliosis severity. These findings may help improve early identification and management of patients at high risk for scoliosis.

## 2 Methods

We retrospectively reviewed patients who underwent cardiac surgery for CHD at our center between 2000 and 2022. A total of 4,148 patients underwent cardiac surgery during the study period. Routine postoperative follow-up at our center included chest radiography. Follow-up duration was calculated from the date of cardiac surgery to the last available chest radiograph. Patients with less than 3 years of postoperative follow-up were excluded. Patients with pre-existing scoliosis before cardiac surgery (*n* = 24), or congenital, neuromuscular, syndromic (e.g., Down syndrome or Marfan syndrome), or other secondary forms of scoliosis (*n* = 6) were excluded. Patients who did not survive the initial hospitalization, lacked complete clinical or radiographic data, were lost to follow-up, or underwent their initial cardiac surgery at another institution were also excluded. Ultimately, 378 patients had adequate postoperative follow-up and complete radiographic data for analysis. Postoperative scoliosis was identified in 39 patients during follow-up. For comparative analysis, 39 postoperative CHD patients without scoliosis were selected from the remaining cohort using propensity score matching (PSM).

Clinical and demographic data were retrospectively collected from medical records, including age, sex, body mass index (BMI), cardiac diagnosis, imaging findings, surgical history, and postoperative complications. Patients were classified as having cyanotic CHD if any primary cardiac diagnosis included a cyanotic lesion. Cyanotic conditions included tricuspid atresia, pulmonary atresia, tetralogy of Fallot, hypoplastic right or left ventricle, and transposition of the great arteries.

Chest radiographs were reviewed to assess Cobb angle, curve pattern, apical vertebral level, thoracic kyphosis, cardiothoracic ratio (CTR), and cardiac morphology. Spinal deformities were measured independently by two evaluators using the Cobb method. Scoliosis was defined as a Cobb angle ≥10°. Thoracic kyphosis was measured between T5 and T12. Cardiac morphology was evaluated by measuring the distances from the thoracic midline to the left and right cardiac borders. These distances were divided by the maximal thoracic transverse diameter to obtain the left and right cardiac lateral extension ratios. For subgroup analyses, patients with scoliosis were stratified according to the median Cobb angle. Patients with a Cobb angle of 10°–27° were classified as the lower Cobb angle group (*n* = 19), whereas those with a Cobb angle >27° were classified as the higher Cobb angle group (*n* = 20). This study was approved by the institutional ethics committee of our hospital.

## 3 Statistical analysis

SPSS 26.0 software (IBM Corporation, Armonk, NY, USA) and R software (version 4.6.1) were used for the statistical analyses. Propensity score matching (PSM) was performed using the nearest-neighbor method at a 1:1 ratio with a caliper width of 0.2. Propensity scores were estimated using sex, age at last follow-up, body mass index (BMI), and follow-up duration to reduce baseline differences between the scoliosis and non-scoliosis groups. Normally distributed continuous variables are presented as the mean ± SD. Comparisons between two groups were performed using the independent-samples t tests, and comparisons among multiple groups were conducted using one-way analysis of variance (ANOVA). Non-normally distributed continuous variables are expressed as the median (interquartile range, IQR). Comparisons between two groups were performed using the Mann–Whitney U test, and comparisons among multiple groups were performed using the Kruskal–Wallis H test. Categorical variables are presented as counts (%), and comparisons were performed using the *χ*^2^ test or Fisher's exact test, as appropriate. The cumulative incidence of scoliosis was estimated using Kaplan–Meier analysis, and differences between groups were assessed using the log-rank test. Associations between Cobb angle and CTR, as well as left and right cardiac lateral extension ratios, were evaluated using Spearman correlation analysis and illustrated with linear regression. Multivariate binary logistic regression analysis was conducted to identify independent risk factors for greater scoliosis severity. A *P* value <0.05 was considered statistically significant.

## 4 Results

A total of 39 patients with scoliosis and 39 matched patients without scoliosis were included in the comparative analysis. The distribution of cardiac structural abnormalities is shown in Table 1. Pulmonary atresia (25.64%) and Ebstein anomaly (20.51%) were the most common cardiac abnormalities in the scoliosis group, whereas single ventricle (28.21%) and Ebstein anomaly (23.08%) were the most common abnormalities in the non-scoliosis group.

**Table 1:** Cardiac structural abnormalities in patients with and without scoliosis.

Cardiac structural abnormality	Non-scoliosis (*n* = 39)	Scoliosis (*n* = 39)
Atrial septal defect	3 (7.69%)	2 (5.13%)
Ventricular septal defect	5 (12.82%)	3 (7.69%)
Tetralogy of Fallot	0 (0.00%)	5 (12.82%)
Double outlet right ventricle	5 (12.82%)	3 (7.69%)
Transposition of the great arteries	0 (0.00%)	1 (2.56%)
Truncus arteriosus	0 (0.00%)	1 (2.56%)
Pulmonary atresia	1 (2.56%)	10 (25.64%)
Absent pulmonary valve	0 (0.00%)	1 (2.56%)
Ebstein anomaly	9 (23.08%)	8 (20.51%)
Tricuspid atresia	3 (7.69%)	3 (7.69%)
Single ventricle	11 (28.21%)	2 (5.13%)
Right ventricular hypoplasia	2 (5.13%)	0 (0.00%)

The clinical characteristics of patients with and without scoliosis are summarized in Table 2. The median follow-up duration for the overall cohort was 12.75 (10.55–18.15) years. No significant differences were observed between the two groups in follow-up duration, age at last follow-up, sex distribution, BMI, EF, or NYHA functional class. Cyanotic CHD was more common in the scoliosis group, with 26 patients (66.67%), compared with 22 patients (56.41%) in the non-scoliosis group, although the difference was not statistically significant (*P* = 0.352). Patients in the scoliosis group tended to undergo their first cardiac surgery at a younger age, with a median age of 2.50 (0.90–5.91) years compared with 3.06 (1.24–6.99) years in the non-scoliosis group, but the difference was not statistically significant (*P* = 0.267). Cardiac surgery before 1 year of age was more common in the scoliosis group, involving 13 patients (33.33%), compared with 6 patients (15.38%) in the non-scoliosis group; however, the difference did not reach statistical significance (*P* = 0.065). The proportion of patients who underwent two or more cardiac surgeries was similar between the two groups [29 [74.36%] vs. 27 [69.23%], *P* = 0.615]. In contrast, patients with scoliosis had a significantly higher CTR than those without scoliosis [55.48 [51.68–58.81] vs. 52.04 [46.62–55.56], *P* = 0.004]. Postoperative complications are summarized in Table 3. The incidences of prolonged mechanical ventilation, prolonged pleural effusion, and sternal wound infection did not differ significantly between the scoliosis and non-scoliosis groups (all *P* > 0.05). No significant differences were observed in late postoperative complications, including heart failure and all-cause mortality (all *P* > 0.05).

**Table 2:** Clinical characteristics of patients with and without scoliosis after cardiac surgery.

Variable	Non-scoliosis (*n* = 39)	Scoliosis (*n* = 39)	Total (*n* = 78)	*P* value
Follow-up (years)	12.92 (10.72–20.21)	12.64 (10.60–16.85)	12.75 (10.55–18.15)	0.849
Age at last follow-up (years)	18.51 ± 8.48	17.90 ± 7.08	18.21 ± 7.77	0.729
Female	21 (53.85%)	23 (58.97%)	44 (56.41%)	0.648
BMI (kg/m^2^)	17.82 (15.59–19.40)	17.72 (15.61–19.57)	17.77 (15.60–19.64)	0.920
CTR (%)	52.04 (46.62–55.56)	55.48 (51.68–58.81)	54.44 (48.86–58.16)	**0** **.** **004**
EF (%)	60.00 (57.50–65.00)	60.00 (60.00–64.50)	60.00 (60.00–65.00)	0.630
NYHA class			0.446	
I	2 (5.13%)	2 (5.13%)	4 (5.13%)	
II	29 (74.36%)	24 (61.54%)	53 (67.95%)	
III	8 (20.51%)	12 (30.77%)	20 (25.64%)	
IV	0 (0.00%)	1 (2.56%)	1 (1.28%)	
Complex CHD	31 (79.49%)	34 (87.18%)	65 (83.33%)	0.362
Cyanotic CHD	22 (56.41%)	26 (66.67%)	48 (61.54%)	0.352
Age at first cardiac surgery (years)	3.06 (1.24–6.99)	2.50 (0.90–5.91)	2.53 (1.06–6.58)	0.267
Cardiac surgery before 1 year of age	6 (15.38%)	13 (33.33%)	19 (24.36%)	0.065
≥2 cardiac surgeries	27 (69.23%)	29 (74.36%)	56 (71.79%)	0.615

Continuous variables are presented as mean ± SD or median (interquartile range). Categorical variables are presented as *n* (%).

BMI, body mass index; CTR, cardiothoracic ratio; EF, ejection fraction; CHD, congenital heart disease; NYHA, New York Heart Association.

**Table 3:** Postoperative complications in patients with and without scoliosis.

Variable	Non-scoliosis (*n* = 39)	Scoliosis (*n* = 39)	*P* value
Early postoperative complications			
Prolonged mechanical ventilation	5 (12.82%)	6 (15.38%)	0.745
Prolonged pleural effusion	6 (15.38%)	4 (10.26%)	0.498
Sternal wound infection	1 (2.56%)	1 (2.56%)	0.999
Late postoperative complications			
Heart failure	8 (20.51%)	13 (33.33%)	0.202
All-cause mortality	1 (2.56%)	3 (7.69%)	0.608

**Figure 1:**
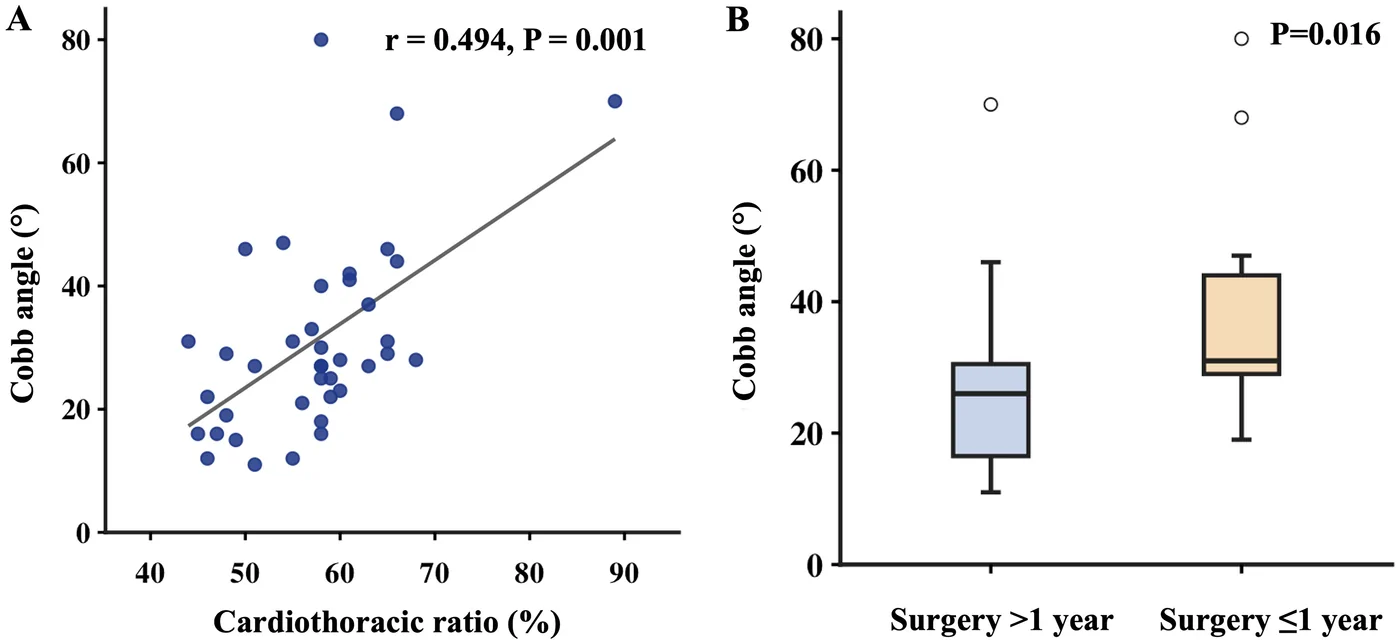
Factors associated with scoliosis severity. **(A)** Correlation between cardiothoracic ratio (CTR) and Cobb angle. **(B)** Comparison of Cobb angle between patients who underwent cardiac surgery at or before 1 year of age and those who underwent surgery after 1 year of age.

To further explore factors associated with scoliosis severity, patients with scoliosis were divided into the lower (*n* = 19) and higher (*n* = 20) Cobb angle groups according to the median Cobb angle. The characteristics of the two groups are summarized in Table 4. Patients with higher Cobb angles had a significantly higher median CTR than those with lower Cobb angles [58.48 [55.39–61.68] vs. 52.85 [49.25–55.88], *P* = 0.003]. In addition, CTR was positively correlated with Cobb angle (r = 0.494, *P* = 0.001; Figure 1A). Cyanotic CHD was more common among patients with higher Cobb angles, affecting 17 patients (85.00%), compared with 9 patients (47.37%) with lower Cobb angles (*P* = 0.019). The median age at scoliosis diagnosis was 10.56 (8.54–13.16) years, with no significant difference between the two groups [11.57 [9.72–14.85] vs. 9.75 [8.18–11.59] years, *P* = 0.112]. The median Cobb angle was 21.00° (16.00–25.00) in the lower Cobb angle group and 38.50° (30.25–46.00) in the higher Cobb angle group. Thoracic kyphosis did not differ significantly between groups (*P* = 0.141). The distribution of scoliosis curve patterns differed significantly between groups (*P* = 0.012). Single thoracic curves were the predominant pattern in the lower Cobb angle group (89.47%), whereas double major curves were more frequently observed in the higher Cobb angle group (30.00%). The main thoracic curve was predominantly right–convex in both groups (78.95% vs. 85.00%, *P* = 0.695). The apical vertebral level was most commonly located at T8-T9, with no significant difference between the two groups (*P* = 0.901).

**Table 4:** Characteristics of scoliosis.

Variable	Lower Cobb angle group (*n* = 19)	Higher Cobb angle group (*n* = 20)	Total (*n* = 39)	*P* value
CTR (%)	52.85 (49.25–55.88)	58.48 (55.39–61.68)	55.48 (51.68–58.81)	**0** **.** **003**
Cyanotic CHD	9 (47.37%)	17 (85.00%)	26 (66.67%)	**0** **.** **019**
Age at scoliosis diagnosis (years)	11.57 (9.72–14.85)	9.75 (8.18–11.59)	10.56 (8.54–13.16)	0.112
Cobb angle (°)	21.00 (16.00–25.00)	38.50 (30.25–46.00)	28.00 (21.00–41.00)	**<0**.**001**
Thoracic kyphosis (°)	16.95 ± 11.80	23.45 ± 15.03	20.28 ± 13.77	0.141
Curve pattern				**0**.**012**
Single thoracic	17 (89.47%)	12 (60.00%)	29 (74.36%)	
Double thoracic	1 (5.26%)	2 (10.00%)	3 (7.69%)	
Double major	0 (0.00%)	6 (30.00%)	6 (15.38%)	
Thoracolumbar	1 (5.26%)	0 (0.00%)	1 (2.56%)	
Right convex main thoracic curve	15 (78.95%)	17 (85.00%)	32 (82.05%)	0.695
Apical vertebra				0.901
T3	1 (5.26%)	1 (5.00%)	2 (5.13%)	
T4	1 (5.26%)	0 (0.00%)	1 (2.56%)	
T5	1 (5.26%)	1 (5.00%)	2 (5.13%)	
T6	1 (5.26%)	1 (5.00%)	2 (5.13%)	
T7	2 (10.53%)	4 (20.00%)	6 (15.38%)	
T8	7 (36.84%)	6 (30.00%)	13 (33.33%)	
T9	6 (31.58%)	7 (35.00%)	13 (33.33%)	

Continuous variables are presented as mean ± SD or median (interquartile range). Categorical variables are presented as *n* (%). Bold values indicate statistical significance (*P* < 0.05).

Surgical characteristics are summarized in Table 5. Cardiac surgery before 1 year of age was more common among patients with higher Cobb angles, with 10 patients (50.00%), compared with 3 patients (15.79%) with lower Cobb angles (*P* = 0.041). Furthermore, patients who underwent cardiac surgery at or before 1 year of age had significantly greater Cobb angles than those who underwent surgery after 1 year of age (*P* = 0.016; Figure 1B). The number of cardiac surgeries differed significantly between groups (*P* = 0.013). Patients in the lower Cobb angle group most commonly underwent two cardiac surgeries (57.89%), whereas those in the higher Cobb angle group most commonly underwent a single cardiac surgery (40.00%). Only patients in the higher Cobb angle group underwent four cardiac surgeries (15.00%). No significant differences were observed between the two groups in the number of MS or thoracotomies (both *P* > 0.05).

**Table 5:** Surgical characteristics of the cohort.

Variable	Lower Cobb angle group (*n* = 19)	Higher Cobb angle group (*n* = 20)	Total (*n* = 39)	*P* value
Cardiac surgery before 1 year	3 (15.79%)	10 (50.00%)	13 (33.33%)	**0.041**
Number of cardiac surgeries				**0.013**
1	2 (10.53%)	8 (40.00%)	10 (25.64%)	
2	11 (57.89%)	5 (25.00%)	16 (41.03%)	
3	6 (31.58%)	4 (20.00%)	10 (25.64%)	
4	0 (0.00%)	3 (15.00%)	3 (7.69%)	
Number of median sternotomies				0.412
0	1 (5.26%)	1 (5.00%)	2 (5.13%)	
1	6 (31.58%)	6 (30.00%)	12 (30.77%)	
2	10 (52.63%)	7 (35.00%)	17 (43.59%)	
3	2 (10.53%)	4 (20.00%)	6 (15.38%)	
4	0 (0.00%)	2 (10.00%)	2 (5.13%)	
Number of thoracotomies				0.176
0	14 (73.68%)	16 (80.00%)	30 (76.92%)	
1	2 (10.53%)	4 (20.00%)	6 (15.38%)	
2	2 (10.53%)	0 (0.00%)	2 (5.13%)	
3	1 (5.26%)	0 (0.00%)	1 (2.56%)	

Categorical variables are presented as *n* (%). Bold values indicate statistical significance (*P* < 0.05).

**Figure 2:**
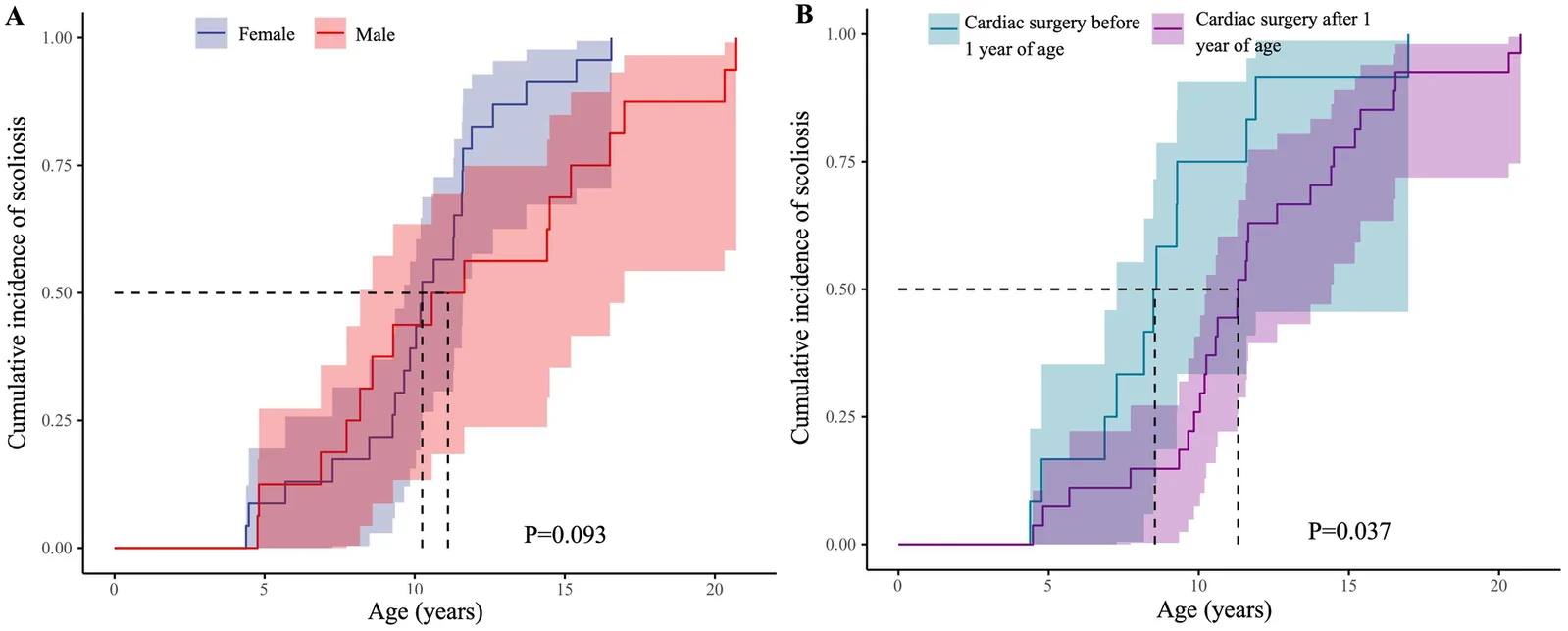
Cumulative incidence of scoliosis according to different clinical subgroups. Kaplan–Meier curves according to sex **(A)**, age at cardiac surgery **(B)**. CHD, congenital heart disease.

The cumulative incidence curves of scoliosis across subgroups are shown in Figure 2. Female patients tended to develop scoliosis earlier than males, although the difference was not significant (Figure 2A, *P* = 0.093). Early cardiac surgery (≤1 year) was associated with earlier onset of scoliosis (Figure 2B, *P* = 0.037).

The results of the logistic regression analysis of factors associated with greater scoliosis severity are shown in Table 6. CTR was dichotomized according to the median value into lower and higher CTR groups. In the univariate analysis, higher CTR, cardiac surgery at or before 1 year of age, and cyanotic CHD were associated with greater scoliosis severity, whereas two or more cardiac surgeries were associated with lower odds of greater scoliosis severity. After adjustment for potential confounding factors, multivariate analysis identified higher CTR as an independent risk factor for greater scoliosis severity (OR 4.79, 95% CI 1.01–22.68, *P* = 0.048).

**Table 6:** Logistic regression analysis of factors associated with greater scoliosis severity.

Variable	Univariate analysis	Multivariate analysis		
	OR (95% CI)	*P* value	OR (95% CI)	*P* value
Age (years)	0.99 (0.90–1.08)	0.776		
Female	1.09 (0.30–3.91)	0.894		
BMI (kg/m^2^)	0.99 (0.85–1.15)	0.866		
Higher CTR	6.53 (1.61–26.47)	0.009	4.79 (1.01–22.68)	0.048
EF (%)	1.03 (0.90–1.18)	0.633		
Complex CHD	5.07 (0.51–50.21)	0.166		
Cardiac surgery before 1 year	5.33 (1.18–24.21)	0.030	4.89 (0.80–29.68)	0.085
≥2 cardiac surgeries	0.18 (0.03–0.98)	0.048		
Cyanotic CHD	6.30 (1.37–28.86)	0.018	2.61 (0.47–14.43)	0.271

OR, odds ratio; CI, confidence interval; CTR, cardiothoracic ratio; EF, ejection fraction; CHD, congenital heart disease.

**Figure 3:**
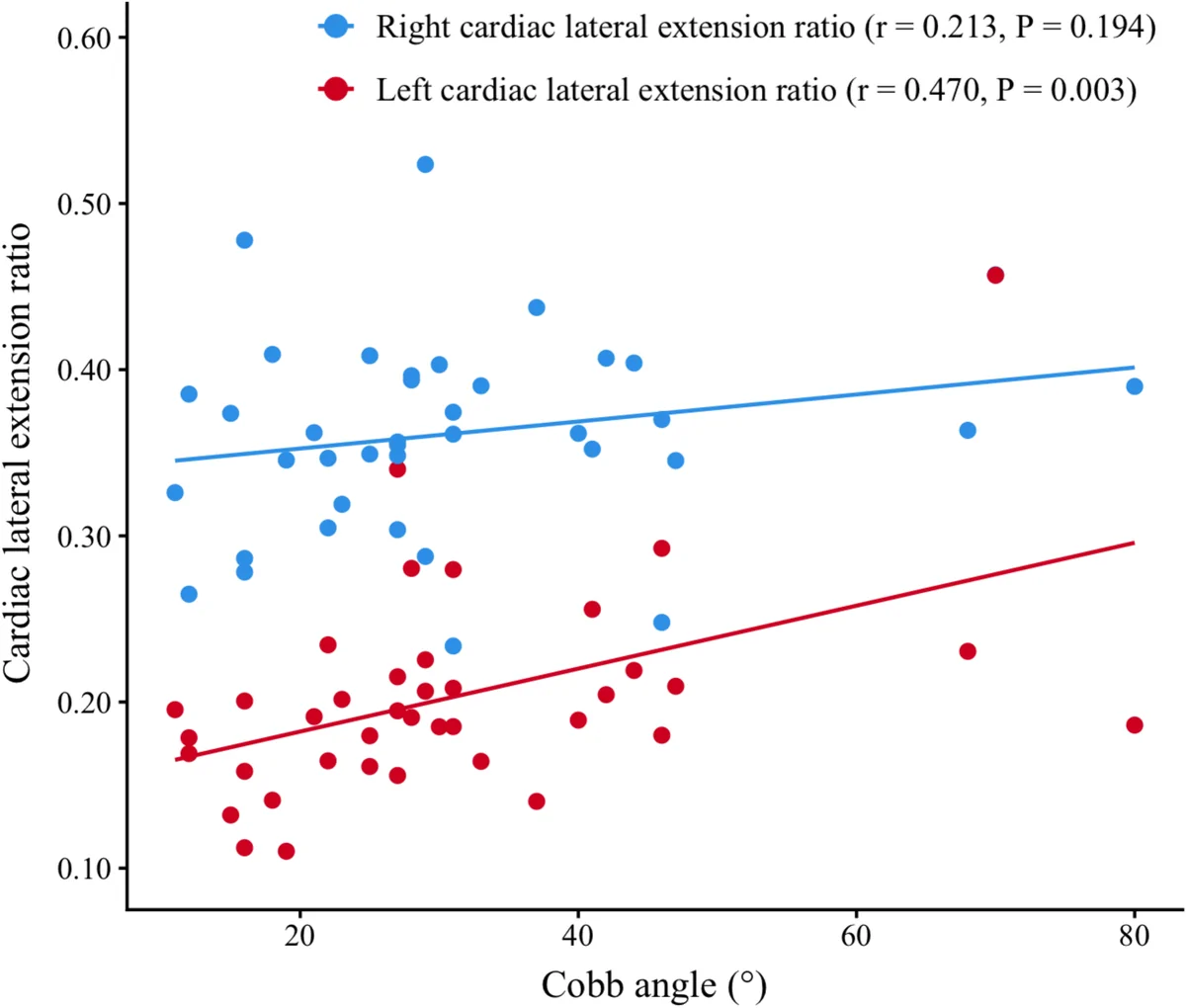
Linear regression analysis between cardiac lateral extension ratios and Cobb angle.

To further explore the relationship between cardiothoracic morphology and scoliosis severity, the left and right cardiac lateral extension ratios were calculated. As shown in Figure 3, the left cardiac lateral extension ratio was positively correlated with Cobb angle (r = 0.470, *P* = 0.003), whereas no significant correlation was observed for the right side (r = 0.213, *P* = 0.194). These findings suggest that greater leftward extension of the cardiac silhouette is associated with greater scoliosis severity.

## 5 Discussion

Increasing evidence suggests an association between CHD and scoliosis. In the present study, we analyzed the clinical characteristics of scoliosis in patients who underwent cardiac surgery for CHD and explored factors associated with scoliosis severity. Our results showed that scoliosis in this population predominantly presented as a right-convex single thoracic curve, and greater scoliosis severity was associated with a higher CTR.

CHD has been considered a potential risk factor for scoliosis. However, the underlying mechanisms remain incompletely understood. They may be related to early embryonic developmental disturbances, hemodynamic alterations secondary to cardiac malformations, and factors related to cardiac surgery (5). From an embryological perspective, both the musculoskeletal and cardiovascular systems originate from the mesoderm, and abnormalities in shared developmental pathways may lead to concurrent defects in these systems (6). Genetic factors may also contribute to this association. Previous studies have suggested that the 22q11.2 deletion may represent a common genetic basis for both CHD and IS (7). Nevertheless, congenital spinal anomalies appear to be relatively uncommon in the CHD population. For example, Reckles et al. (8) reported only one case (<1%) of congenital scoliosis among 377 patients with CHD. Similarly, another study including 498 children with CHD identified congenital vertebral anomalies in only five patients (1%) (9). In our initial screening, only 6 patients were found to have congenital spinal anomalies. These findings suggest that primary structural spinal anomalies are unlikely to be the main mechanism underlying scoliosis development in patients with CHD.

Cardiac surgery has been considered an important contributor to scoliosis in patients with CHD. Several studies have reported a significantly higher prevalence of scoliosis in children with CHD after thoracic surgery (4, 10). A large cohort study by Mery et al. (11) demonstrated that thoracic surgery significantly increased the risk of scoliosis and moderate-to-severe scoliosis, with 10-year prevalence rates of 12% and 3%, respectively. Notably, this association does not appear to depend on the underlying disease that initially required thoracic surgery, as similar findings have been reported in patients without CHD (12, 13). These observations suggest that thoracic surgery itself may contribute to scoliosis development. One possible mechanism is that thoracic surgery may disrupt the normal growth of the thoracic cage and spine, leading to asymmetric rib growth and subsequent spinal curvature (5).

Surgical treatment for CHD primarily involves MS and thoracotomy, or a combination of both approaches. Previous studies have shown that scoliosis can occur after either surgical approach (3, 14). A previous study suggested that when both procedures are performed, the thoracic spine may experience a “double impact,” potentially increasing the risk of spinal deformity (4). Regarding MS, Kawakami et al. (15) reported that the majority of scoliosis patients (54 of 74) had previously undergone MS. Ruiz-Iban et al. (16) followed patients who underwent MS for at least 9 years and found that the prevalence of scoliosis on standard posteroanterior chest radiographs in adulthood reached 34%. The mechanism may involve disruption of normal sternal and thoracic growth. Longitudinal division of the immature sternum may alter the normal growth pattern of the sternum and thoracic cage, thereby affecting thoracic biomechanics and promoting the development of scoliosis or sagittal plane deformities (17). In addition, thoracic retraction during surgery may injure the costovertebral joints, intercostal nerves, or rib epiphyses, which has been shown in experimental models to induce secondary scoliosis (18). For thoracotomy, structural damage to the thoracic cage may disrupt the mechanical balance of the spine, gradually leading to spinal curvature (19). However, the relationship between the direction of the scoliotic curve and the side of the surgical incision remains controversial. Some studies in patients with esophageal atresia have reported that the scoliotic curve tends to deviate toward the side opposite the surgical approach (12). In contrast, Van Biezen et al. (13) observed that scoliosis in patients with coarctation of the aorta more commonly curved toward the side of the thoracotomy. Furthermore, whether cardiac surgery accelerates scoliosis progression remains debated. A recent large retrospective study suggested that thoracotomy or MS does not significantly accelerate scoliosis progression (14). Similarly, Reckles et al. (8) found no clear association between the type or location of surgical incision and the development of IS in patients with CHD. Consistent with these findings, the present study also did not observe a significant association between incision type or location and scoliosis severity. Therefore, the precise role of cardiac surgery in the development of scoliosis requires further investigation.

Previous studies have suggested that the timing of cardiac surgery may be associated with scoliosis development in patients with CHD. Ruiz-Iban et al. (16) reported that the prevalence of scoliosis reached 46% in patients who underwent cardiac surgery before 18 months of age, and surgical age was the factor most strongly associated with scoliosis development. Similarly, Kaito et al. (9) reported that the prevalence of scoliosis exceeded 40% among children who underwent CHD surgery during the first year of life. This phenomenon may be explained by the fact that the thoracic cage and spine undergo rapid growth during infancy. At this stage, the thoracic and spinal structures are not yet fully mature, and surgery-related mechanical trauma may interfere with the normal development of the thoracic cage and spine, thereby increasing the risk of scoliosis after CHD surgery (20). In our cohort, patients who underwent cardiac surgery before 1 year of age tended to have more severe scoliosis and were diagnosed at a younger age. However, cardiac surgery before 1 year of age was not independently associated with greater scoliosis severity after adjustment for potential confounding factors, suggesting that the observed association may be influenced by other clinical characteristics, such as cardiac morphology or disease severity. Therefore, the contribution of surgical timing to scoliosis development remains uncertain and warrants further investigation.

The impact of the number of surgeries on scoliosis remains controversial. In our study, the number of cardiac surgeries did not appear to be clearly associated with the occurrence of scoliosis. However, we noted that some patients with greater scoliosis severity had undergone multiple cardiac surgeries, suggesting that repeated surgical procedures may contribute to cumulative mechanical injury to the thoracic cage and spine, thereby affecting normal thoracic and spinal growth. Similarly, a case–control study by Kawamura et al. (2) reported that severe scoliosis was more common in patients who had undergone multiple thoracic surgeries.

Among patients with scoliosis, females accounted for 58.97%, and the median age at scoliosis diagnosis was 10.56 years, which is consistent with previous reports (4, 9). Although the difference did not reach statistical significance, female patients tended to be diagnosed with scoliosis at a younger age than male patients. These findings suggest that scoliosis is often detected during adolescence, a period characterized by rapid spinal growth and increased susceptibility to biomechanical imbalance (21). In addition, the more pronounced growth acceleration and hormonal changes during female puberty may influence bone metabolism and spinal growth, thereby contributing to the development and progression of scoliosis. Radiographically, single thoracic curves were the predominant pattern, with the apical vertebra most frequently located at T8-T9, which is consistent with previous studies of scoliosis in patients with CHD (2).

Another important finding of this study was the significant association between the CTR and scoliosis severity, suggesting that cardiac enlargement may play an important role in scoliosis progression. Kaito et al. (9) similarly reported that cardiomegaly was the only predictor of a Cobb angle greater than 45°. Although Kawamura et al. (2) did not observe a significant difference in CTR between patients with and without scoliosis, they reported that CTR increased in parallel with scoliosis severity, further supporting a potential relationship between cardiac enlargement and curve progression. The underlying mechanism remains unclear. One possible explanation is that cardiac enlargement may alter the mechanical balance within the thoracic cavity, potentially affecting normal thoracic and spinal development and promoting the progression of scoliosis. In addition, ventricular pulsation may produce asymmetric forces on the thoracic wall, gradually contributing to lateral spinal deviation during growth (9). In further analysis, we found that the leftward extension of the cardiac silhouette was associated with scoliosis severity. Given the predominantly left-sided anatomical position of the heart, enlargement of left-sided cardiac structures may exert greater mechanical influence on the thoracic cavity, leading to asymmetric loading of the thoracic cage and increased lateral stress on the spine. However, the observational nature of this study precludes any inference of causality, and the underlying mechanism requires further investigation. Taken together, these findings suggest that CTR may serve as a potential radiographic marker for risk stratification in patients with scoliosis after CHD surgery.

These findings suggest the importance of early surveillance and timely intervention in patients at higher risk of developing scoliosis after cardiac surgery for CHD. This study has several limitations. First, this was a single-center retrospective study with a relatively small sample size, which may limit the generalizability of the findings. Moreover, the retrospective design precludes causal inference between cardiac surgery and the subsequent development of scoliosis, and the possibility that some patients had idiopathic scoliosis independent of cardiac surgery cannot be completely excluded. Second, the analysis was limited to patients with long-term follow-up, which may have introduced selection bias. Not all patients had reached skeletal maturity at the end of follow-up, potentially leading to an underestimation of the true incidence. Third, because scoliosis was identified at the time of radiographic diagnosis, the recorded age reflected the age at diagnosis rather than the actual onset of scoliosis. Future multicenter prospective studies with larger cohorts and longer follow-up are warranted to further clarify the mechanisms underlying scoliosis development in patients with CHD.

## 6 Conclusions

In conclusion, scoliosis after cardiac surgery for CHD most commonly presented as a right-convex single thoracic curve. A higher CTR was associated with the presence of scoliosis and independently associated with greater scoliosis severity. These findings suggest that CTR may serve as a simple radiographic marker for risk stratification, and long-term spinal surveillance should be considered in children with CHD after cardiac surgery.

## Data Availability

The raw data supporting the conclusions of this article will be made available by the authors, without undue reservation.
